# Structure of the UspA1 protein fragment from *Moraxella catarrhalis* responsible for C3d binding

**DOI:** 10.1016/j.jsb.2019.08.002

**Published:** 2019-11-01

**Authors:** Kornelia M. Mikula, Robert Kolodziejczyk, Adrian Goldman

**Affiliations:** a Molecular and Integrative Biosciences, Faculty of Biological and Environmental Sciences, University of Helsinki, Helsinki, Finland; b Astbury Centre for Structural Molecular Biology, School of Biomedical Sciences, University of Leeds, Leeds, UK

**Keywords:** CEACAM1, carcinoembryonic antigen-related cell adhesion molecule 1, CV, column volume, h, hydrophobic, IPTG, isopropyl β-d-1-thiogalactopyranoside, LB, Luria broth, PBS, phosphate buffered saline, p, polar, SAXS, small-angle X-ray scattering, TAA, trimeric autotransporter adhesin, Usps, ubiquitous surface proteins, *Moraxella catarrhalis*, UspA1, TAAs, C3d protein, complement system, X-ray structure

## Abstract

The gram-negative bacterium *Moraxella catarrhalis* infects humans exclusively, causing various respiratory tract diseases, including acute otitis media in children, septicaemia or meningitis in adults, and pneumonia in the elderly. To do so, *M. catarrhalis* expresses virulence factors facilitating its entry and survival in the host. Among them are the ubiquitous surface proteins (Usps): A1, A2, and A2H, which all belong to the trimeric autotransporter adhesin family. They bind extracellular matrix molecules and inhibit the classical and alternative pathways of the complement cascade by recruiting complement regulators C3d and C4b binding protein.

Here, we report the 2.5 Å resolution X-ray structure of UspA1^299–452^, which previous work had suggested contained the canonical C3d binding site found in both UspA1 and UspA2. We show that this fragment of the passenger domain contains part of the long neck domain (residues 299–336) and a fragment of the stalk (residues 337–452). The coiled-coil stalk is left-handed, with 7 polar residues from each chain facing the core and coordinating chloride ions or water molecules. Despite the previous reports of tight binding in serum-based assays, we were not able to demonstrate binding between C3d and UspA1^299–452^ using ELISA or biolayer interferometry, and the two proteins run separately on size-exclusion chromatography. Microscale thermophoresis suggested that the dissociation constant was 140.5 ± 8.4 μM. We therefore suggest that full-length proteins or other additional factors are important in UspA1-C3d interactions. Other molecules on the bacterial surface or present in serum may enhance binding of those two molecules.

## Highlights

• UspA1^299–452^ is a left-handed coiled-coil structure that follows TAA rules.
• Structure of UspA1^299–452^ contains part of the long neck domain and of the stalk.
• UspA1-C3d binding does not saturate at C3d physiological concentrations.
• The binding constant as measured by thermophoresis is at least 140 μM.
• Full-length proteins or other factors are important for UspA1-C3d interactions.

## Graphical abstract

**Figure:**
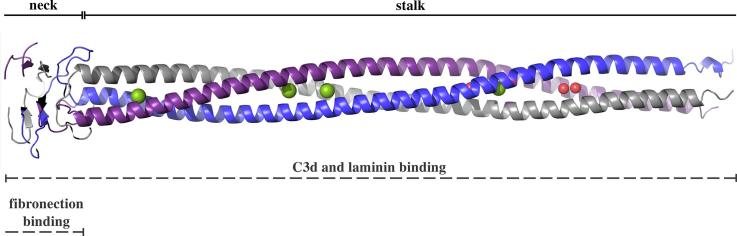

## 1 Introduction

*Moraxella catarrhalis* is a gram-negative bacterium that infects humans exclusively (de Vries et al., 2009). Up to 80% of children under 2 years old carry *M. catarrhalis*: this rate drops to 10% for older children and to 5% for healthy adults, and increases again in the elderly (Aebi, 2011). Although for many years it was considered to be only a commensal, *M. catarrhalis* is now classed as a pathogen. After *Streptococcus pneumonia* and *Heamophilus influenza*, it is the third most common pathogen causing acute otitis media in children (Verduin et al., 2002). In adults with chronic obstructive pulmonary disease, *M. catarrhalis* induces not only upper but also lower respiratory tract infections, causing infections as severe as septicaemia, meningitis or endocarditis in immunocompromized patients; and pneumonia in the elderly (Hassan, 2013, Verduin et al., 2002).

To cause infections, *M. catarrhalis* expresses different adhesion macromolecules that act as virulence factors in key aspects of bacteria pathogenesis. The most important ones are outer membrane proteins such as *M. catarrhalis* adherence protein (McaP), protein CD, *M. catarrhalis* filamentous Hag (FHA)-like proteins (Mha proteins), *M. catarrhalis* immunoglobulin D (IgD) binding protein/hemagglutein (MID/Hag), and ubiquitous surface proteins (Usps) (reviewed in de Vries et al., 2009). The “ubiquitous surface protein” (Usp) family consists of three proteins: UspA1 (88 kDa), UspA2 (62 kDa), and UspA2H (92 kDa). UspA2H is a hybrid of the first two; it contains a UspA1-like N-terminal domain and a UspA2-like C-terminal domain (Aebi et al., 1997, Lafontaine et al., 2000). *M. catarrhalis* attaches to epithelial cells *via* UspA1, which binds carcinoembryonic antigen-related cell adhesion molecule 1 (CEACAM1) (Hill and Virji, 2003), and as a consequence suppresses the human inflammatory response (Slevogt et al., 2008). UspA1 also binds extracellular matrix proteins laminin (Tan et al., 2006) and fibronectin (Agnew et al., 2011, Tan et al., 2005), whereas UspA2 binds preferentially to laminin (Tan et al., 2006), fibronectin (Tan et al., 2005), and vitronectin (McMichael et al., 1998). Another important function associated with UspA proteins is serum resistance. Both UspA1 and UspA2/A2H have been proposed to bind the C3d domain of C3, inhibiting both the classical and alternative pathways of the complement cascade (Hallström et al., 2011, Nordström et al., 2005). Furthermore, UspA1 and UspA2 appear to bind to the complement inhibitor C4b binding protein (C4BP) in a dose dependent manner (Nordström et al., 2004). Finally, UspA proteins block generation of the opsonin C3a, which may result in decreased inflammatory reactions (Hallström et al., 2011). This last would be consistent with binding C3d (Lambris et al., 2008).

**Fig. 1:**
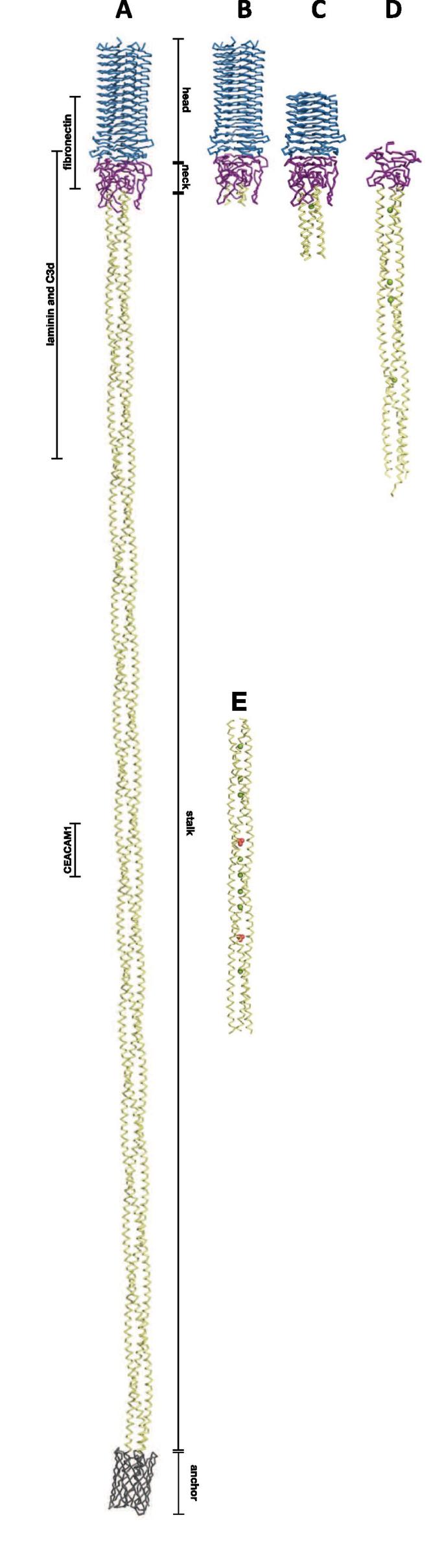
Comparison of UspA1 structures from *Moraxella catarrhalis.* (A) Modelled structure of full-length UspA1 with four differently coloured and labelled regions, starting from N-terminus: head (blue), neck (purple), coiled-coil stalk (yellow) and trans-membrane β-barrel (grey). Sites for ligand binding marked on the left-hand side: fibronectin (165–318), laminin and C3d (299–452), CEACAM1 (578–597). Structures of different solved UspA1 fragments (B) UspA1^42–345^ (3PR7) (Agnew et al., 2011); (C) UspA1^153–366^ (3NTN) (Agnew et al., 2011); (D) UspA1^299–452^ (6QP4) solved in this study; (E) UspA1^527–665^ (2QIH) (Conners et al., 2008); Cl^−^ ions in green and PO_4_^3−^ in red.

UspA proteins belong to the trimeric autotransporter adhesin (TAA) family. TAAs are anchored in the bacterial outer membrane by a 12-stranded β-barrel (the translocation domain) comprised of four strands from each monomer, from where the passenger domain is exposed to the extracellular environment. The passenger domain consists of an N-terminal β-strand head domain followed by the neck domain and a coiled-coil stalk (Bassler et al., 2015) (Fig. 1A). Although no full-length structure of UspA1 is available, there are structures of three UspA1 fragments. Two structures (3NTN and 3PR7) (Agnew et al., 2011) together give a fragment comprising UspA1^42–366^, containing the head, the neck, and 33 amino acids of the stalk domain (Fig. 1B, C). The head domain consists of 14-to-16 residue repeats placed parallel to each other forming a trimeric left-handed parallel β-roll, first identified in YadA (Nummelin et al., 2004). The neck (region 276–334) is a positively charged region of the UspA1 structure forming large loops (Agnew et al., 2011); it belongs to the long neck type (Hartmann et al., 2012) as found in SadA (2YO2, 2YNZ) (Hartmann et al., 2012) or BpaA (3LAA) (Edwards et al., 2010). The structure of part of the stalk of UspA1 (UspA1^527–665^) has been solved (2QIH) (Conners et al., 2008) (Fig. 1E). It is supposed to bind CEACAM1 (see below). It reveals a continuous left-handed trimeric coiled-coil stalk with, as expected, an underwound periodicity of 3.5 residues per turn, characteristic for TAA proteins (Conners et al., 2008). Taken together, currently available crystallographic structures of the UspA1 molecule cover 464 out of 821 amino acids, not much more than 50%.

So far, no high-resolution structure of UspA1 in complex with its ligands is available. However, based on small-angle X-ray scattering (SAXS), molecular modelling and mutagenesis studies, models have been proposed of UspA1-ligand complexes (Agnew et al., 2011, Conners et al., 2008). The CEACAM1 binding site appears to be within the segment 578–597 of the stalk domain containing His584, which is the only potentially charged residue in that region surrounded by hydrophobic residues. Mutagenesis and binding studies strongly indicated that Ala568, 588 and 509, Leu583, and Met586 are crucial residues involved in CEACAM1 binding (Conners et al., 2008). In addition, SAXS measurements suggested that UspA1 bends upon CEACAM1 binding (Conners et al., 2008). Similarly SAXS and binding data suggested that fibronectin (FnIII_12-15_ fragment) binds at the base of the β-roll head domain and causes bending of UspA1 at the interaction site (Agnew et al., 2011). Finally, the Riesbeck group proposed, based on ELISA binding assays between C3d and truncated UspA1 fragments, that C3d binds the coiled-coil stalk in the 299–452 region (Hallström et al., 2011).

In this study, we present the crystallographic structure of UspA1^299–452^, which contains the putative C3d binding site (Hallström et al., 2011). We performed multiple binding studies between recombinant C3d and UspA1^299452^, but were not able to demonstrate saturation binding; the *K*_d_ appears to be 140.5 ± 8.4 μM, which is not consistent with physiological concentrations of C3.

## 2 Materials and methods

### 2.1 Cloning of protein constructs

We amplified UspA1^299–452^ from the UspA1 gene (Gen bank: AAD43465) of *Moraxella catarrhalis* strain ATCC 25238 (BC5) as a template using the following primers: forward GCCG**CATATG**AAAACTGGTAATGGTACTGTATCT containing an *Nde*I restriction site (in bold), and reverse GGCG**AAGCTT**GCTGCCGCGCGGCACCAGATCAATGAGGCGACCGCTTA containing a thrombin cleavage site (underlined) and a *Hind*III restriction site (in bold). The PCR product was digested with *Nde*I and *Hind*III restriction enzymes (Thermo Fisher Scientific) and ligated into pET22b(+) (Novagen) plasmid using T4 ligase (Thermo Fisher Scientific).

For C3d, we used a construct previously received by our laboratory (Isenman et al., 2010, Kajander et al., 2011), recloned it to the pET22b vector by restriction-free (RF) cloning using the following primers: forward: CTTTAAGAAGGAGATATACATATGCATCATCATCATCATCACAGCAGCGGCGAAAACCTGTATTTTCAGAGCGA and reverse: TCGGGCTTTGTTAGCAGCCGGATCTCAGCGGCTGGGCAGTTGGAGGGACAC. Secondly, we reversed the E1153A point mutation using the primers: forward: GTTCTCATCTCGCTGCAGGAAGCTAAAGATATTTGCGAG and reverse: CTCGCAAATATCTTTAGCTTCCTGCAGCGAGATGAGAAC. The last step was to remove the free Cys1010, changing it to Ala (C1010A) using primers: forward GACCCCCTCGGGCGCGGGGGAACAGAAC, and reverse GTTCTGTTCCCCCGCGCCCGAGGGGGTC. Point mutations were introduced by QuickChange® Site-Directed Mutagenesis (Stratagene, BMC Biotechnol, (Liu and Naismith, 2008)).

### 2.2 Protein expression

For expression, positive plasmids carrying C3d or UspA constructs were transformed into BL21(DE3) *E. coli* chemically competent cells, plated on LB (Luria Broth)-agar plates supplemented with 50 μg/ml ampicillin and incubated overnight (O/N) at 37 °C. For large-scale expression of UspA1^299–452^, clones from the plate were inoculated into 5 ml LB media supplemented with 100 μg/ml ampicillin, grown O/N at 24 °C, diluted into 500 ml of fresh LB with 100 μg/ml ampicillin and then incubated at 37 °C, 220 rpm shaking. Protein production was induced with 1 mM isopropyl β-d-1-thiogalactopyranoside (IPTG). For large-scale expression of C3d, clones from the plate were inoculated into 5 ml LB media supplemented with 100 μg/ml ampicillin and grown O/N at 18 °C. They were then diluted into 50 ml of fresh LB with 100 μg/ml ampicillin and incubated at 24 °C, 220 rpm shaking for 4 h, followed by another dilution into 500 ml of fresh LB with 100 μg/ml ampicillin, grown at 24 °C until they reached OD = 0.8 and protein production was induced with 1 mM IPTG. For both proteins, expression was continued for 4 h, after which bacteria were collected by centrifugation for 20 min at 5000×*g* at 4 °C. The supernatant was discarded and pellets resuspended, for UspA1^299–452^ in 10 ml of buffer A (20 mM Na_x_H_x_PO_4_, pH 8.0, 500 mM NaCl, 10 mM imidazole), and for C3d in 10 ml of buffer B (20 mM Na_x_H_x_PO_4_, pH 8.0, 300 mM NaCl, 10 mM imidazole). Proteins were either purified directly (see below) or cells were flash-frozen in liquid nitrogen and stored at −80 °C for later use.

### 2.3 Protein purification

Cells were disrupted using the Emusiflex-C3 (Avestin) for 10 min at 1500 psi, then centrifuged for 45 min at 18000×g at 4 °C; the supernatant (around 10 ml) was transferred to a 50 ml Falcon tube and incubated with 2 ml of NiNTA beads (Qiagen) (previously equilibrated with buffer A or buffer B as appropriate) for 30 min with gentle shaking and loaded onto a 20 ml gravity flow column (BioRad). Beads were washed with 10 column volumes (CV) of buffer A (UspA1^299–452^) or buffer B (C3d). For UspA1^299–452^, there was an additional washing step with 5 CV of 20 mM Na_x_H_x_PO_4_, pH 8.0, 500 mM NaCl, 50 mM imidazole. The protein of interest was then eluted with 3 CV of 20 mM Na_x_H_x_PO_4_, pH 8.0, 250 mM imidazole and either 300 mM (C3d) or 500 mM (UspA1^299–452^) NaCl. Elutions containing the protein of interest were pooled and loaded onto Superdex 200 (GE Healthcare) size exclusion chromatography column with 1 × PBS (phosphate buffered saline) for C3d purification and 20 mM HEPES, pH 8.0, 500 mM NaCl for UspA1^299–452^ purification. Fractions containing the protein of interest were concentrated with Amicon® Ultra 4 ml concentration filter with a molecular mass cutoff of 10 kDa, flash-frozen in liquid nitrogen and stored at −80 °C.

### 2.4 Binding studies

The binding of C3d to UspA1^299–452^ was studied by thermophoresis using a Monolith NT.115 (NanoTemper Technologies GmbH, Germany). Before the experiment, both proteins were exchanged into 0.5 × PBS (70 mM NaCl, 1.4 mM KCl, 5 mM Na_2_HPO_4_, 0.9 mM KH_2_PO_4_), which was used as the reaction buffer during the entire experiment. Preparation, labelling, dilutions and initial measurements were performed according to the manufacturer’s instructions (Monolith NT^TM^ His-Tag Labelling Kit RED-tris-NTA). The concentration of fluorescently labelled UspA1^299–452^ was kept constant at 50 nM and the C3d was titrated from 23 nM to 750 μM. Measurements were performed with 70% LED (Light-Emitting Diode) and 40% MST (MicroScale Thermophoresis) power. Sample preparation and measurements were repeated three times for statistical relevance. The difference in normalized fluorescence (ΔF) was plotted against concentration of unlabelled C3d and the *K*_d_ calculated using equations provided in the software for data analysis from thermophoretic measurements (NanoTemper Technologies GmbH, Germany). Final graph was prepared using Prism (GraphPad) program.

### 2.5 Crystallization and data collection

For crystallization trials, UspA1^299–452^ and C3d were concentrated to 2.4 mg/ml and 10.7 mg/ml respectively using Amicon® Ultra 4 ml concentration filters with a molecular mass cut-off of 10 kDa; as UspA1 is a trimer, proteins were mixed in 1 to 3 molar ratio (UspA1:C3d) and crystallization drops of 200 nl (100 nl of protein solution and 100 nl of well solution) were set up in 96-well MRC (Molecular Dimensions) crystallization plates using a mosquito LCP® (TTP Labtech, UK). Helsinki Random I and II (HRI and HRII) screens (http://www.biocenter.helsinki.fi/bi/xray/automation/services.html), our local modifications of the classic sparse matrix screens (Cudney et al., 1994), yielded initial hits from conditions: HRI: 30% MPD, 0.1 M Na-Cacodylate, pH 6.5, 0.2 M Mg-Acetate; and 18% PEG8000, 0.1 M Na-Cacodylate, pH 6.5, 0.2 M Zn-Acetate; HRII: 3.4 M Hexanediol, 0.1 M Tris, pH 8.5, 0.02 M MgCl_2_. Grid screens prepared manually around the initial hits were used to optimise crystal growth and diffraction. For final optimization, hanging drops were set up manually (2 μl protein solution + 2 μl well solution) using the following grid screen: 0.5–4.0 M 1,6-Hexanediol, 0.1 M Tris-HCl, pH 8.5, 20 mM MgCl_2_. The 2.9 M 1,6-Hexanediol present in the well solution also served as a cryoprotectant when flash freezing crystals in liquid nitrogen. Data were collected on the ADSC Quantum Q315r detector at beamline ID14-4 at the European Synchrotron Research Facility (ESRF) in Grenoble, France.

### 2.6 Data processing, structure solution and refinement

Images were processed to 2 Å resolution in space group *P*2_1_ using XDS (Kabsch, 2010), the quality of the data assessed with phenix.xtriage (Adams et al., 2010) (Table 1) and data anisotropy analysed using the UCLA-DOE Diffraction Anisotropy Server (https://services.mbi.ucla.edu/anisoscale/) (Strong et al., 2006). Diffraction data were reprocessed to 2.5 Å based on F/σ values for each crystal direction obtained from anisotropy analysis. The structure was solved by molecular replacement using Molrep (Vagin and Teplyakov, 2010) in the CCP4 package (Winn et al., 2011) with the structure of UspA1^165–366^ (PDB: 3PR7) (Agnew et al., 2011) as a model. Model building was done in Coot (Emsley et al., 2010) followed by refinements in Refmac5 (Murshudov et al., 2011, Skubak et al., 2004) and phenix.refine (Adams et al., 2010). Finally, structure quality was assessed using the MolProbity webserver (Chen et al., 2010) and the Phenix software package (Adams et al., 2010).

**Table 1:** Crystallographic table for UspA1^299–452^. Diffraction data and refinement statistics.

Wavelength	0.939270 Å		
Space group (number)	*P*2_1_		
Unit cell (Å)	a = 63.64, b = 44.57, c = 128.44 β = 92.07°		

*Diffraction data*			
	Overall	Inner Shell	Outer Shell

Resolution (Å)	2.50	46.01–7.40	2.65–2.50
R_meas_ (%)	7.6	2.7	36.2
No. of reflections measured	91,893	3314	14,443
No. of reflections unique	25,116	961	3950
<I/σI>	16.66	41.11	4.74
Completeness (%)	98.4	91.3	96.8
Multiplicity	3.7	3.4	3.6

*Refinement*			
R value (%)	21.22		
R_free_ (%)	27.20		

*Final structure*			
R.m.s. deviations from ideal			
Bond lengths (Å)	0.014		
Bond angles (°)	1.492		
Chirality (°)	0.063		
Planarity (°)	0.008		
Dihedral (°)	8.968		
Ramachandran statistics (%)			
Favoured	96.96		
Allowed	1.95		
Outliers	0.22		
Rotamer outliers	0.0		
Content of asymmetric unit			
Protein atoms	3526		
Solvent molecules	199		
Chloride ions	4		
Zinc ions	2		
1,2-Hexanediol molecules	14		
B-factors (average in Å^2^)			
for protein	62.5		
for solvent	46.1		

### 2.7 Structure analysis

The structure was analysed using the daTAA server (https://toolkit.tuebingen.mpg.de/dataa) for TAA structure analysis (Szczesny and Lupas, 2008), and HBPlot (http://dept.phy.bme.hu/virtuadrug/hbplot/bin/infopage.php) to analyse secondary and tertiary protein structure (Bikadi et al., 2007, McDonald and Thornton, 1994). The coiled-coil characteristics were evaluated using programs TWISTER (Strelkov and Burkhard, 2002) and SOCKET (http://coiledcoils.chm.bris.ac.uk/socket/) (Walshaw and Woolfson, 2001). A model of full-length UspA1 was built using CCBuilder (Wood et al., 2014). Figures of UspA structures were prepared using PyMol (Molecular Graphics System, Version 1.8 Schrödinger, LLC.).

## 3 Results

### 3.1 Crystals of UspA1^299–452^ show anisotropy

After data processing with XDS (Kabsch, 2010) we truncated the processed data to 2.0 Å resolution, based on CC(1/2) values exceeding 50%. However, after solving the structure and starting refinement we noticed that R_free_ remained around 30–35%, which was unexpectedly high. One possible explanation was that C3d was still missing from the model, as it was present in the crystallization solution. However, we did not see any unbuilt density that would indicate that UspA1^299–452^ crystallized in complex with C3d, and the V_m_ was 3.54 Å^3^ Da^−1^, which was not consistent with the presence of another molecule. The second option was data anisotropy, which was confirmed by the UCLA-DOE LAB Diffraction Anisotropy Server (Strong et al., 2006). Plots of F/σ for the three reciprocal space axes a*, b*, and c* against resolution give the maximum resolution for which F/σ exceeds 3 in each direction. For our dataset, the resolution limits along a* and b* were 2.0 Å, but only 2.5 Å along c*. As a next step the server performs ellipsoidal truncation, anisotropy scaling and applies negative isotropic B-factor correction. The automatically generated corrected structure factors, however, resulted in overall completeness lower than 90%; 35% between 2.10 and 2.05 Å, 68% between 2.29 and 2.22 Å. We therefore chose to cut our data manually to 2.5 Å. Using these data we solved the structure and refined it to acceptable R factors for the resolution: R_free_ = 27.20% and R_work_ = 21.22% (Table 1).

### 3.2 UspA1^299–452^ is a coiled-coil structure that follows TAA rules

The asymmetric unit of the crystal contained one trimer of UspA1^299–452^, as is typical of a trimeric autotransporter adhesin. It consists of part of the neck domain (residues 299–336) and the stalk (residues 337–452). The neck domain of UspA1^299–452^ is a long neck containing, towards its end, the characteristic DAVN motif (Bassler et al., 2015) that mediates the transition from the left-handed parallel β-roll headgroup to the α-helical stalk. The neck ends with the typical QL sequence: Leu337, the last residue of the neck, is also the first residue of the stalk domain that follows. The stalk domain is a left-handed coiled-coil built mostly from heptad repeats with the typical arrangement of repeating hydrophobic (h) amino acids separated by polar (p) residues in the seven-residue *abcdefg* pattern hpphppp*.* There are, however, two disruptions from the heptad pattern: after Gly396 and Gly442 in the *d* position, there is a Leu that occurs structurally at position *a*, instead of the canonical polar amino acid at position *e* to complete the *abcdefg* heptad pattern. In addition to Leu, there are another three amino acids (LeuAspLeu) inserted in these sites making them an 11-residue pattern. This changes the periodicity of the coiled-coil from heptad (7-residue pattern) to hendecad (11-residue pattern), which is consistent with the daTAA server predictions (Szczesny and Lupas, 2008).

**Fig. 2:**
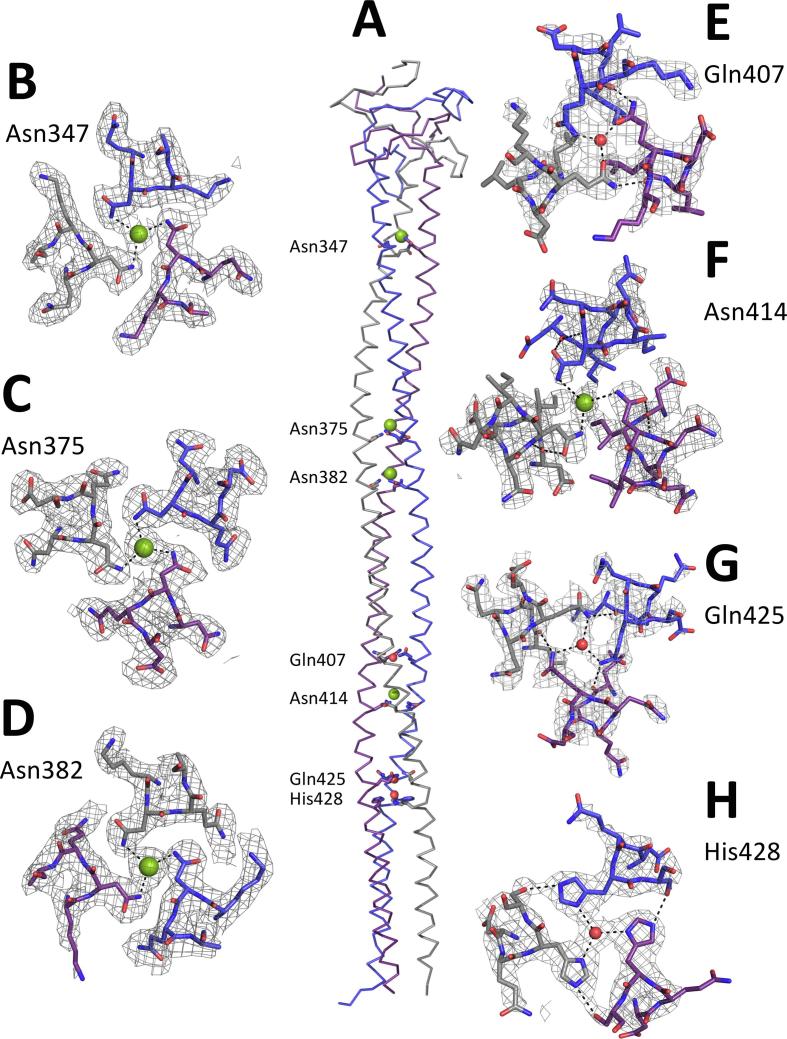
Structure of UspA1^299–452^. Model of UspA1^299–452^ structure solved in this study, neck and coiled-coil domains; chain A in blue, chain B in grey, and chain C in purple, chloride ions in green and water molecules in red present in cavity of coiled-coil; (A) side view; bottom view of the centre of the coiled-coil core with non-typical amino acids in *a* and *d* positions (B-H): coordination of Cl^−^ ions by Asn side chains in *d* position: (B) Asn345, (C) Asn375, (D) Asn382 and (F) Asn414; coordination of H_2_O molecules by (E) Gln407 and (H) His428 in *d* position and (G) Gln425 in *a* position.

Typically, hydrophobic residues in positions *a* and *d* form the core of the coiled-coil (Bassler et al., 2015). In our structure the majority of the residues in position *a* are hydrophobic: 16 out of 17 amino acids are either Ile, Leu or Val, with one amino acid in position *a* being polar, Gln425. On the other hand, 9 of 17 of the *d* positions are occupied by Leu, 2 of 17 by Gly where the heptad pattern changes (described above), while the other six positions are occupied by the polar amino acids Asn (347, 375, 382 and 414), Gln407, and His428 (Fig. 2). The side chains of Asn347, 375, 382 and 414 in the *d* positions face the central core of the trimer and the amide nitrogens of their side chains coordinate chloride ions, forming characteristic N@*d* layers (Fig. 2B–D and F) (Hartmann et al., 2009); the distances of those interactions range from 2.90 to 3.61 Å, as expected (Table 2) (Carugo, 2014). Cl^−^ coordinated by amide nitrogens of Asn414, in contrast to Cl^−^ coordinated by other Asn, have only 50% occupancy, as there was strong negative (F_o_-F_c_) density after refinement with 100% occupancy (more details in Discussion). Asn347 forms a VxxNxxx pattern, whereas Asn375, 382 and 414 form an IxxNxxx pattern.

**Table 2:** Distances and B-factors of interactions between polar amino acids occupying *a* and *d* positions and molecules they coordinate or other amino acids. B stands for B-factor in Å^2^; d stands for distance in Å.

Polar amino acid in position *a* or *d*	Chain A	Chain B	Chain C	Interaction partner
Asn 347 (Nδ2)	B: 29.59 d: 3.25	B: 29.24 d: 3.16	B: 32.92 d: 3.30	Cl B: 35.15
Asn 375 (Nδ2)	B: 43.40 d: 2.95	B: 47.48 d: 3.29	B: 43.56 d: 2.90	Cl B: 36.01
Asn 382 (Nδ2)	B: 50.55 d: 3.14	B: 54.25 d: 3.18	B: 48.81 d: 3.29	Cl B: 44.43

Gln 407	Nε2 B: 79.30 d: 2.35	Oε1 B: 74.72 d: 2.40	Oε1 B: 77.10 d: 2.35	H_2_O B: 65.96
Gln 407	Oε1 B: 99.24 d: 3.19	Nε2 B: 70.42 d: 2.83	Nε2 B: 88.37 d: 2.64	Leu 404 (O) A-B, B-C, C-A

Asn 414 (Nδ2)	B: 79.26 d: 3.61	B: 83.77 d: 2.93	B: 77.74 d: 3.43	Cl (50% occupancy) B: 56.00

Gln 425 (Nε2)	B: 81.66 d: 2.50	B: 89.69 d: 2.84	B: 91.47 d: 2.98	H_2_O B: 74.64
Gln 425 (Nε2)	d: 3.10	d: 3.31	d: 2.90	Leu 421 (O) A-C, B-A, C-B

His 428 (Nδ1)	B: 85.62 d: 3.03	B: 79.07 d: 2.61	B: 84.00 d: 2.76	H_2_O B: 69.15
His 428 (Nε2)	B: 89.75 d: 2.75	B: 79.24 d: 2.65	B: 81.14 d: 2.58	Ser 429 (Oγ) A-B, B-C, C-A

Gln407 and 425 in *d* positions coordinate water molecules in the centre of trimeric core (Fig. 2E, G). Gln407 interacts with water molecule through Nε2 of chain A and by Oε1 of chains B and C. This mixed orientation of the Gln407 side chains is caused by additional interactions of Nε2 from chains B and C with oxygen of Leu404 from a neighbouring chain (Fig. 2E) (Table 2). Hydrogen bonds formed by these interactions are 2.6–2.8 Å in length, whereas Oε1 Gln407 of chain A and Leu404 from chain B are 3.2 Å apart (Table 2). In the case of Gln425, the geometry of the interactions is different. The side chains of Gln425 are arranged clockwise (looking from bottom to top), which is in contrast to the anti-clockwise arrangement of Gln407 (Fig. 2E, G). Side chains of all three Gln425 are oriented with Nε1 towards the trimer core where they coordinate water molecule. This orientation is additionally stabilized by second interactions of Gln425 Nε1 with Leu421 oxygen from neighbouring chain (Fig. 2G) (Table 2). Finally, His428 coordinates a water molecule through Nδ2. The core facing orientation of His428 is stabilized by interactions of Nε2 with Oγ of the following Ser429 from the neighbouring chain (Fig. 2H) (Table 2).

### 3.3 Detailed geometry of the UspA1 stalk

The solved UspA1^299–452^ is a parallel, left-handed, 3-stranded coiled-coil. The angles between the helices are between 5.6 and 6.9° calculated using SOCKET (Walshaw and Woolfson, 2001). There are 74 type 4 ‘knobs into holes’ interactions with packing angles between 37.8 and 74.4°. Angles were calculated between the Cα-Cβ bond vector of the knob residue and the Cα-Cα vector between the two residues that form the sides of the hole (Harbury et al., 1994).

**Fig. 3:**
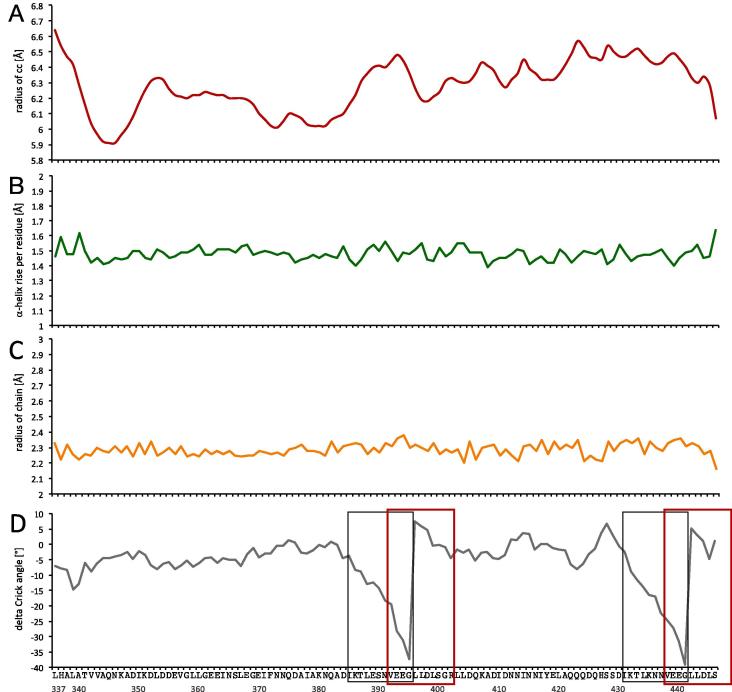
Analysis of coiled-coil parameters of UspA1^299–452^. Analysis of the stalk (Leu337-Ser447) of UspA1^299–452^ using program TWISTER shows features typical for coiled-coil protein. (A) A radius of coiled-coil in Å; (B) a rise of an α-helix in Å per residue; (C) a radius of helix in Å calculated for chain A; (D) Crick angle shift (°) calculated for chain A based on model TAA structure (1GCM) (Harbury et al., 1994). The grey box indicates two regions with heptad distortion; the red box indicates hendecad.

TWISTER (Strelkov and Burkhard, 2002) showed that the average α-helix rise per residue is 1.51 Å (Fig. 3B). It appears that the long neck at the N-terminus, with the characteristic 120° rotation of the monomers (such that neck A is structurally above helix B, neck B above helix C *etc*) keeps the coiled-coil together, whereas the truncated C-terminus has no structural motif keeping three chains coiled, which would be supplied by the C-terminal β-barrel anchor in the full-length protein. For that reason, we checked the parameters of the coiled-coil to make sure it is not distorted. The radius of coiled-coil and helix vary from 5.91 to 6.64 Å and 2.20 to 2.38 Å respectively (Fig. 3 A and C). The variations of the coiled-coil radius are the result of many factors. The transition from the neck to the stalk domain (starting at L337) causes the biggest drop in the coiled-coil radius from 6.64 Å to 5.91 Å at N347, forming tight interactions by coordination of the Cl^−^ ion. Disruptions of heptad pattern are another apparent cause of increase of the coiled-coil radius. The N*@d* layers appear to keep the coiled-coil tight, as can be seen in local radius minima (Fig. 3A). The Crick angle describes the orientation of a residue in relation to the coiled-coil axis, whereas its shift (Δϕ) expresses the difference between angular values of the amino acid of the given structure and the model structure. To calculate the Crick angle shift we first calculated ideal angles for positions *a – g* using the GCN4 leucine zipper core mutant (pdbe code: 1GCM) as a model TAA structure (Harbury et al., 1994). We then subtracted those values from angles calculated for our structure for each position. The Crick angle Δ varies between +3.6° and −8.9° and significantly deviates in two regions of the UspA1 structure (Fig. 3D) due to the change in periodicity to hendecads after Gly396 and Gly442, where the pattern is not *abcdefgabcdefg*, but *abcdabcdefg* (Fig. 3D, red box). The deviation starts with a gradual increase in Δϕ from Ile386 in position *a* through the whole heptad to Glu396 in position *d* of the following heptad for which Δϕ reaches a maximum and returns to average values for the following residue, Leu397 in position *a*. We observe the same pattern for another region of the UspA1^299–452^, which starts with Ile432 and reaches maximum for Gly442. Previously solved structures of UspA1 did not show any other periodicity than the heptad pattern.

### 3.4 Binding between C3d and UspA1^299–452^ is weaker than expected.

**Fig. 4:**
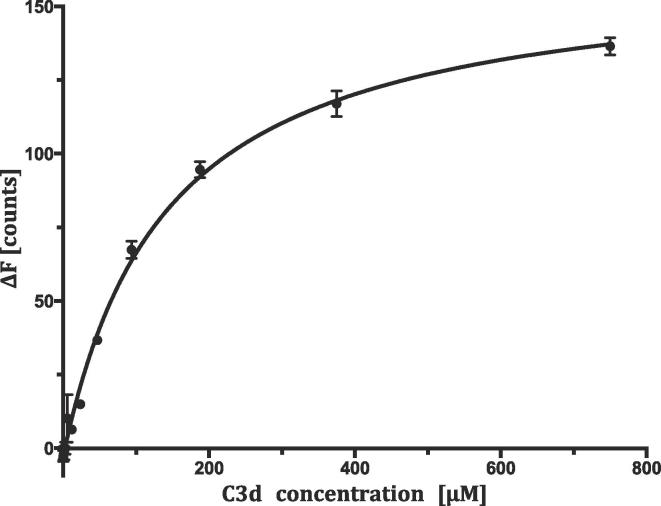
Binding between C3d and UspA1^299–452^. Studies of interactions between fluorescently labelled UspA1^299–452^ and the unlabelled C3d. The UspA1^299–452^ concentration is kept constant at 50 nM and the C3d is titrated from around 23 nM to 750 μM. The difference in normalized fluorescence (ΔF) is plotted against concentration of unlabelled C3d. Raw data from the measurements were imported to Prism where all calculations were done. The calculated *K*_d_ equals 140.5 ± 8.4 μM. Error bars represent standard error of *n* = 3 measurements.

For the binding and crystallization studies, we introduced two point mutations into C3d, E1153A and C1010A. The first one was to revert to the wild-type C3d sequence and the second removed the free Cys residue; E1153 and C1010 form the active thioester payload of C3 and we preferred to have the charge of the glutamate but not the potential for aggregation of an unpaired cysteine (Isenman et al., 2010, Kajander et al., 2011). UspA1 fragment (amino acids 299–452) was the same as the fragment previously designed and reported to bind C3d by Hallström et al. (2011). Our attempts to crystalize C3d in complex with UspA1^299–452^, which was previously reported to bind C3d (Hallström et al., 2011), were unsuccessful. Moreover, in contrast to previous reports (Hallström et al., 2011), we were not able to demonstrate binding between C3d and UspA1^299–452^ using ELISA or biolayer interferometry, and the two proteins ran separately on size-exclusion chromatography and blue native gel (data not shown), suggesting that the binding is weak. This is also consistent with the fact that the crystals, grown at 1:3 molar ratio of UspA1:C3d and with a C3d concentration of 10.7 mg/ml (0.32 mM) contained no C3d at all. We decided to investigate binding in solution using thermophoresis. This method does not require immobilization of either of the proteins to measure binding, so there would be no interference from surface effects. Recombinantly produced and purified C3d was concentrated to 750 μM, which was the highest we could achieve without aggregation. UspA1^299–452^ was concentrated to 200 nM, as needed for the labelling. In the thermophoresis experiment, we titrated 50 nM labelled UspA1^299–452^ with increasing concentrations of unlabelled C3d from 22.9 nM up to 750 μM and measured the fluorescence. We could not reach full binding saturation (Fig. 4) even with the maximum concentration of C3d. Changes in fluorescence were normalized, plotted against C3d concentration, and a binding constant of 140.5 ± 8.4 μM was calculated (Fig. 4).

## 4 Discussion

The UspA1^299–452^ we solved in this study is the fourth high-resolution structure of part of UspA1. Two of the structures solved previously, UspA1^42–345^ (3PR7) and UspA1^153–366^ (3NTN) (Agnew et al., 2011), also contain the neck domain and beginning of the stalk. Our structure, however, contains a longer fragment of the stalk, which encompasses the putative C3d binding site. The fourth structure, UspA1^527–665^ (2QIH), is of a fragment of the stalk closer to C-terminal membrane anchor (Conners et al., 2008) (Fig. 1).

As expected for a TAA, we observed polar amino acids in positions *a* (Gln) and *d* (Asn, Gln, and His). This is unlike canonical coiled-coils, where amino acids occupying positions *a* and *d* build the core of a trimer and are always hydrophobic (Boyken et al., 2016). In the UspA1^153–366^ and UspA1^527–665^ structures, Asn occupy positions *d* with their side chains facing the core of a trimer and in each case coordinating a chloride ion. UspA1^527–665^ has eight N@*d* layers, and additionally three His in either the *a* or *d* positions. Two of the His coordinate phosphate ions and the other a water molecule (Conners et al., 2008). This is similar to our structure, where His428 in position *d* coordinates a water molecule. This histidine is part of the most frequent heptad motif QxxHxxx in TAAs with a hydrophilic core (Hartmann et al., 2009).

Many other TAA structures have N@*d* layers in their coiled-coil stalks, such as EibD (2XQH) (Leo et al., 2011), SadA (2YO0) (Hartmann et al., 2009), and AtaA (3WPA, 3WPO, 3WQA) (Koiwai et al., 2016). In the above cases, Asn side chains in N@*d* layers coordinate Cl ions. In the UspA^299–452^ structure that we report here, four Asn residues occupy positions *d* (Asn347, 375, 382 and 414). The first three form standard N@*d* layers, coordinating Cl^−^. In the case of Asn414, however, evidence for Cl^−^ coordination is not that obvious. First of all, the density is weak and is consistent with approximately 50% occupancy: using 100% occupancy results in strong negative (F_o_-F_c_) density (data not shown). Furthermore, the concentration of Cl ions in the solution is at least 200 mM (crystallisation solution contains 20 mM KCl, 100 mM Tris-HCl pH 8.5 ≈ 30% acid form; protein solutions contain >300 mM NaCl; see methods) so half-occupancy implies a binding constant of 200 mM.

On the other hand, when modelling water molecule at 100% occupancy in place of Cl^−^ we satisfy the (F_o_-F_c_) density map but orientation of Asn side chains remains as for Cl ion coordination. Moreover, coordination distances ranging from 2.94 to 3.61 Å would argue against coordination of water molecule. In both cases B factors 56.00 and 59.91 Å^2^ for Cl^−^ and water, respectively are lower than coordinating Asn residues (ranging from 77.74 to 83.77 Å^2^). Finally, R and Rfree factors for structure with Cl^−^ equal 21.22 and 27.20%, respectively and are very similar to the ones (R = 21.28 and Rfree = 27.26%) for the structure with water molecule instead. Resolution of the structure, which is 2.5 Å, does not allow us to resolve this issue. Following the rule of N@*d* layers and lack of known exception to it, we decided to refine the structure with Asn414 coordinating Cl ion with half-occupancy. Nonetheless, it is worth pointing out that this binding site is *not* occupied at physiological concentrations of chloride ion, which are typically around 100 mM in blood plasma.

In addition, we found two Gln facing the core of the trimer, one in position *a* and another in position *d,* each coordinating a water molecule. In none of the published UspA1 structures has Gln been reported to occupy core positions. However, in the light of all the TAA structures and predictions, Gln is one of the most common polar amino acids found in positions *a* or *d* (Bassler et al., 2015).

Furthermore, as the UspA1^299–452^ solved in this study was previously reported to bind complement protein C3 (Nordström et al., 2005) and in particular to its cleavage product C3d (Hallström et al., 2011), we performed a series of binding experiments between UspA1^299–452^ and C3d. We were not able to reproduce the ELISA results (Hallström et al., 2011) using their approach though, of course, not their reagents. We were also unable to demonstrate complex formation between UspA1^299–452^ and C3d by size exclusion chromatography or biolayer interferometry. ELISA and biolayer interferometry methods require, however, surface immobilization of one of the proteins, which could result in a geometrically unfavourable orientation of the molecule where access to the binding site might be limited or completely blocked. This would lead to underestimation of binding affinities of the two proteins. There might also be other surface effects, such as unfavourable interactions of the ligand protein with the surface. If the binding was weak, such effects might make it unobservable.

Thermophoresis, we reasoned, is performed in solution and so allows both proteins to interact freely with each other. Despite this we were not able to obtain full saturation of the binding at the maximal possible C3d concentration of 750 μM. Curve fitting of the binding data nonetheless allowed us to calculate a *K*_d_ of 140.5 ± 8.4 μM. This is about twenty times higher than the physiological concentrations of C3 in the serum, which ranges from 4.3 to 8.5 μM. The *K*_d_ is also ten times weaker than that measured between full-length UspA1 and C3met by Nordström et al. (2005).

What might explain this discrepancy? One possibility is that Nordström et al. performed their measurements using full-length UspA1 passenger domain, and even though later Hallström et al. narrowed down the C3 and UspA1 interactions to C3d and UspA1^299–452^, they were only able to show it by ELISA (Hallström et al., 2011, Nordström et al., 2005). Secondly, some of their experiments were performed on whole bacterial cells expressing UspA with serum or serum-purified C3d, not on a biochemically-pure system. Our *in vitro* experiments measure for the first time the interaction of C3d with UspA1^299–452^ without any confounding factors. We therefore suggest that additional factors may be important in UspA1-C3d interactions. Other parts of the UspA1 passenger domain might be also involved in interactions with C3d, or fragments of C3 molecules that are cleaved off in C3d take part in stabilizing the interactions. In addition, other molecules on the bacterial surface or present in serum could enhance binding of those two molecules. Studies of the interaction of full-length UspA1 with C3d should, however, now be possible with the new generation of electron microscopes.

## 5 Deposition of coordinates

Coordinates and structure factors of the UspA1^299–452^ crystal structure were deposited with Protein Data Bank in Europe (PDBe); accession code 6QP4.
